# Supplementary material for: Livestock Depredation by Large Carnivores and Human–Wildlife Conflict in Two Districts of Balochistan Province, Pakistan
Source: Animals (Basel). 2024 Apr 4;14(7):1104. doi: 10.3390/ani14071104 (PMC11010808; doi:10.3390/ani14071104)
Supplement: Supplementary file 1 [file animals-14-01104-s001.zip › animals-2892032-supplementary.pdf]

Model Summary

Multiple R-squared	0.6331
Adjusted R-squared	0.6119
F-statistic	29.84
P-value	2.2e-16

This multiple R-squared value indicates that approximately 63.31% of the variance in perceptions about wildlife is explained by the variables included in your model. The adjusted R-squared adjusts for the number of predictors in the model and is slightly lower than the multiple R-squared. The F-statistic tests the overall significance of the model. With a very low p-value, we can reject the null hypothesis that all coefficients are equal to zero, suggesting that the model is statistically significant.

Interpretation of Coefficients

Intercept: The intercept term represents the estimated perception when all other predictor variables are zero. Here, it's approximately 0.9999.

Education (factor): The coefficients for different education levels represent the change in perception compared to the reference level (Education level 1). For example, individuals with Education level 2 have a perception increase of approximately 0.0141 compared to Education level 1.

Economic Loss: As the economic loss due to wildlife increases (e.g., from 25000 to 75000), perceptions become increasingly negative. For instance, compared to no economic loss, individuals experiencing a loss of 75000 show a perception decrease of approximately 0.7476.

Occupation (factor): The coefficients for different occupations indicate how perceptions vary across different occupations compared to the reference occupation. However, none of these coefficients are statistically significant at conventional levels.

Total Livestock Owned (T.o): Each additional unit of total livestock owned is associated with a decrease in perception of approximately 0.0037.

Interaction Effects

There are interaction effects between Education and Economic Loss. These coefficients represent the combined effect of Education and Economic Loss on perception. For example, the coefficient for "Education 2: Economic Loss 25000" suggests that individuals with Education level 2 and experiencing a loss of 25000 have a perception decrease of approximately 0.2558 compared to the reference category.

Interpretation

The model indicates that education, economic losses due to wildlife, and total livestock owned significantly influence perceptions about wildlife. Higher levels of education are associated with slightly more positive perceptions. Economic losses due to wildlife have a strong negative impact on perceptions, with higher losses leading to more negative perceptions. Occupation does not appear to have a significant independent effect on perceptions in this model. Total livestock owned also has a slight negative association with perceptions. Interaction effects between education and economic loss suggest that the combined effect of these variables can further influence perceptions.

Limitations:

While the model explains a significant portion of the variance in perceptions, there may be other unmeasured factors influencing perceptions about wildlife. Causal interpretations should be made cautiously, as the analysis is based on observational data. The presence of missing data may have influenced the results, as evidenced by the deletion of 10 observations.
